# Techno-economic evaluation of integrated first- and second-generation ethanol production from grain and straw

**DOI:** 10.1186/s13068-015-0423-8

**Published:** 2016-01-04

**Authors:** Elisabeth Joelsson, Borbála Erdei, Mats Galbe, Ola Wallberg

**Affiliations:** Department of Chemical Engineering, Lund University, P.O. Box 124, 22100 Lund, Sweden

**Keywords:** Techno-economic, Simulations, First- and second-generation, Ethanol, Biorefinery, Wheat, Grain, Straw, Biogas, DDGS

## Abstract

**Background:**

Integration of first- and second-generation ethanol production can facilitate the introduction of second-generation lignocellulosic ethanol production. Consolidation of the second-generation with the first-generation process can potentially reduce the downstream processing cost for the second-generation process as well as providing the first-generation process with energy. This study presents novel experimental results from integrated first- and second-generation ethanol production from grain and wheat straw in a process development unit. The results were used in techno-economic evaluations to investigate the feasibility of the plant, in which the main co-products were distiller’s dried grains with solubles and biogas.

**Results:**

An overall glucose to ethanol yield, of 81 % of the theoretical, based on glucose available in the raw material, was achieved in the experiments. A positive net present value was found for all the base case scenarios and the minimal ethanol selling price varied between 0.45 and 0.53 EUR/L ethanol. The revenue increased with combined xylose and glucose fermentation and biogas upgrading to vehicle fuel quality. A decrease in the biogas yield from 80 to 60 % also largely affects the net present value. The energy efficiency for the energy content in products available for sale compared with the incoming energy content varied from 74 to 80 %.

**Conclusions:**

One of the two main configurations can be chosen when designing an integrated first- and second-generation ethanol production plant from grain and straw: that producing biogas or that producing distiller’s dried grains with solubles from the xylose sugars. The choice depends mainly on the local market and prices for distiller’s dried grains with solubles and biogas, since the prices for both co-products have fluctuated a great deal in recent years. In the current study, however, distiller’s dried grains with solubles were found to be a more promising co-product than biogas, if the biogas was not upgraded to vehicle fuel quality. It was also concluded that additional experimental data from biogas production using first- and second-generation substrates are required to obtain improved economic evaluations.

## Background

Most bioethanol is currently produced from sugar- and starch-containing materials, such as sugar cane, corn, and wheat grain [1]. Ethanol production from these easily accessible sugars is usually referred to as first-generation (1G) production. However, the use of these crops has become the subject of debate as they are food crops, and it has been argued that they can be put to better use as food. The use of lignocellulosic materials, such as agricultural residues, forest materials, and dedicated crops, referred to as second-generation (2G) production, is therefore being promoted. However, lignocellulosic materials have a more recalcitrant structure and a different carbohydrate composition than the materials used in 1G ethanol production; in addition the residual material generated in the 2G process differs from the 1G process. The more complex 2G process affects the overall design and usually increases the cost for 2G ethanol production, e.g., the requirement for pretreatment and handling of various solid materials.

Integration of 1G and 2G ethanol production can offer a means of reducing the cost of producing 2G bioethanol while the technology matures, and helping to establish also 2G ethanol production. Furthermore, integration of the sugar-rich material to 2G fermentation results in a higher ethanol concentration in the broth without the need to increase the solid content, which would also increase inhibition. A higher ethanol concentration can decrease the cost of downstream processing, such as distillation, in the 2G plant, while at the same time supplying the 1G plant with heat and electricity produced from the residual solid material from the 2G plant. Integration can be achieved either by designing a completely new combined plant, or by installing a 2G unit at an existing 1G plant.

The production of wheat, one of the largest starch-grain products in Europe, with a production of 230 million tons in 2013 [2], also results in large amounts of residual straw. Some of this straw should be left on the field as soil conditioner; however, the rest could be used for ethanol production. The integration of a 1G and a 2G plant-producing ethanol from grain and straw is thus of considerable interest. Integration is possible at several stages in the process, from directly after pretreatment, to the downstream processes, for example, in the distillation or evaporation steps. Thus, several different process configurations are possible. In the present study, integration in the fermentation step is considered. This will increase the ethanol concentration in the broth, thus reducing the energy demand in distillation compared to a 2G stand-alone plant. Moreover, the water consumption in the process can also be decreased by utilizing liquid from the 2G process to dilute the broth in the fermentation step of the 1G process.

Techno-economic evaluations have recently been performed for integrated 1G and 2G (1G + 2G) ethanol production from sugar cane and lignocellulosic residues, such as bagasse and trash [3–6]. Several studies on the simulation of ethanol production have also been reported, for example, from starch (corn with and without corn stover) [7–9], corn stover [10–12], and other lignocellulosic agricultural residues [13, 14]. However, to the best of the authors’ knowledge, no techno-economic evaluations of the integration of 1G + 2G ethanol production from grain and wheat straw have been performed, although evaluations of 1G + 2G ethanol production in various biorefinery systems have been carried out for wheat grain and wheat straw separately. Various alternatives for the production of value-added co-products and the improvement of the process in a 1G wheat-based plant have then been investigated. Arifeen et al. [15] studied the extraction of gluten, yeast, and husk for co-product production, as well as yeast cell recycling and on-site enzyme production, in order to improve the process. Sadhukhan et al. [16] investigated the potential to extract arabinoxylan from the bran fraction as a co-product. The effect of the conversion of the stillage to biogas instead of distillers dried grain with solubles (DDGS) on the energy consumption of the 1G plant has been described by Pfeffer et al. [17] and, more recently, by Rajendran et al. [18]. Studies on the 2G production of ethanol from wheat straw have focused on heat integration and exergy analysis [19, 20], downstream processing of the stillage for anaerobic digestion or evaporation [21], and techno-economic evaluations of a number of process alternatives for various co-products [22].

In the techno-economic evaluations of integrated 1G + 2G ethanol production that have been performed, mainly sugar cane and bagasse were the substrates considered. These processes differ from that with grain and straw in the composition of the raw material and the co-products. One of the main co-products of ethanol production from sugar cane and bagasse is electricity, while in the case of grain and straw, DDGS can be produced as a co-product from the considerable amount of protein in the grain. Thus the co-products are attractive in two completely different markets.

The present study was performed to evaluate the feasibility of ethanol production in an integrated 1G + 2G plant using grains and wheat straw as the raw materials. Experimental trials were first carried out in a process development unit with 30-L reactors to verify the fermentation results obtained previously on lab scale using a mixture of wheat and barley grain meal (WBG) and wheat straw by Erdei et al. [23–26]. The results were then used in computer simulations of different cases to investigate how the production of DDGS and other co-products would affect the economy of the plant. Aspen Plus was used for the simulations since it can handle both material and energy balances, and provides dimensioning data for the equipment, which are needed in the economic evaluations. The economic evaluations were performed with Aspen Process Economic Analyzer (APEA) supplemented with data from vendors’ quotations. In addition, Aspen Plus is useful for performing sensitivity analysis when experimental results are insufficient or unreliable. An example of this is the anaerobic digestion system, which has not been extensively explored concerning the use of substrates from 1G + 2G ethanol production. In this case, by varying the yield of biogas the effect on the NPV can be estimated.

## Results and discussion

### Experimental results

**Fig. 1:**
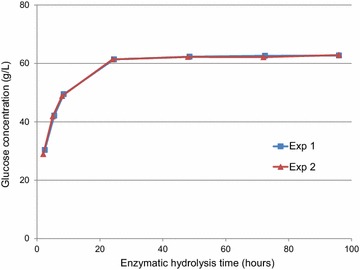
Results of enzymatic hydrolysis (EH) of the steam-pretreated wheat straw (SPWS)

**Fig. 2:**
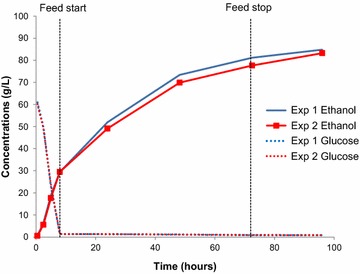
Fermentation of the hydrolyzed steam-pretreated wheat straw (SPWS) and the wheat and barley grain (WBG)

The results of enzymatic hydrolysis (EH) of the steam-pretreated wheat straw (SPWS), and the fermentation of the SPWS and the WBG are shown in Figs. 1 and 2. Duplicate experiments, denoted as Exp 1 and Exp 2, were performed in both EH and ftermentation. The liquid fraction of the hydrolyzed SPWS was first added to the fermenter, and after 8 h the feeding of the saccharified WBG started.

The final glucose concentration in EH was 62.8 ± 0.2 g/L, and was reached after only 24 h, corresponding to an average glucose yield of 87 ± 0 % of the theoretical (Fig. 1). Therefore, the hydrolysis time was set to 24 h in the Aspen Plus simulations. The final xylose concentration was 10.5 ± 0.1 g/L, corresponding to a xylose yield of 84.8 ± 0 % of the theoretical in both cases (data not shown).

Figure 2 shows the ethanol and glucose concentrations during 96 h of fermentation. The ethanol production rate was highest during the first 8 h, 3.7 g/L ethanol per hour, and then decreased significantly to an average of 1.3 g/L ethanol per hour. This was due to the somewhat low feeding rate, (200 ml/h corresponding to 50 g glucose/h) which did not allow faster production as no glucose accumulated in the broth. The ethanol concentration however increased even after the feed was stopped (after 72 h), which is likely due to the presence of intracellular metabolites. Glucose is often taken up faster than ethanol is produced, which will result in intracellular glucose and ethanol accumulation that cannot be detected, when the broth is analyzed. The final ethanol concentration was 84.0 ± 1.1 g/L after 96 h. The overall process ethanol yield based on the amount of glucose available in the raw material was calculated to be 81.3 %. In the simulations, the overall ethanol yield was 79.7 % since it was assumed that some glucose was utilized for yeast cultivation.

### Simulations

Seven cases were simulated in the present study. The cases consisted of a 1G stand-alone plant (1G) followed by six integrated 1G + 2G plants. In the three first cases (1G, E1, and B1) only glucose fermentation was considered. In E1, concentration by evaporation of the thin stillage, generated in the solid–liquid separation step after distillation, was modeled. The concentrated solution was then re-mixed with the solids generated in the solid–liquid separation step after the distillation. This mixture was subsequently transferred to the dryer. In B1, the thin stillage was transferred to anaerobic digestion instead of being concentrated. In case C5E1 and C5B1, glucose and xylose fermentation was modeled for the E1 and B1 configurations. The last two cases UB1 and UC5B1 were based on B1 and C5B1, respectively; however, the biogas was in these cases upgraded to vehicle fuel quality. A more detailed description of the cases is presented in the section “Methods” under “Case description”.

### Energy

Table 1 gives the energy and mass flow of the incoming materials and outgoing products for the seven cases simulated, using the lower heating value (LHV) for the different materials based on their heat of combustion. In case 1G, E1, B1, and UB1 only glucose (C6) fermentation to ethanol was assumed in the simulations, and in the rest of the cases xylose (C5) fermentation to ethanol also was assumed in the simulations.

**Table 1:** The mass and energy flows of incoming materials and outgoing products for the seven cases

Case	1G		E1		B1		C5E1		C5B1		UB1		UC5B1	
Unit	tons/h	MW	tons/h	MW	tons/h	MW	tons/h	MW	tons/h	MW	tons/h	MW	tons/h	MW
Inputs														
Raw material, 1G	55	240	45	198	45	198	45	198	45	198	45	198	45	198
Raw material, 2G	0	0	23	116	23	116	23	116	23	116	23	116	23	116
Methane produced and used in plant	0	2	3	37	1	10	2	21	1	14	1	10	1	14
Natural gas (methane) purchased	4	58	0	1	0	0	1	20	0	0	0	0	0	0
Electricity produced and used in plant	–	4	–	5	–	6	–	5	–	6	–	8	–	7
Fresh water	122	0	141	0	141	0	134	0	135	0	141	0	135	0
Harvesting and transportation	–	7	–	9	–	9	–	9	–	9	–	9	–	9
Outputs														
Ethanol	20	147	20	147	20	147	22	167	22	167	20	147	22	167
Methane (sold)	0	0	0	0	4	53	0	0	3	35	4	53	3	35
Methane produced total	0	2	3	37	5	63	2	21	4	49	5	63	4	49
DDGS (dry)	19	85	21	91	9	38	19	83	9	37	9	38	9	37
Carbon dioxide	20	0	20	0	20	0	23	0	22	0	20	0	22	0
Electricity, total	–	14	–	14	–	9	–	14	–	9	–	9	–	9
Electricity, sold	–	10	–	8	–	3	–	9	–	4	–	0	–	2

**Fig. 3:**
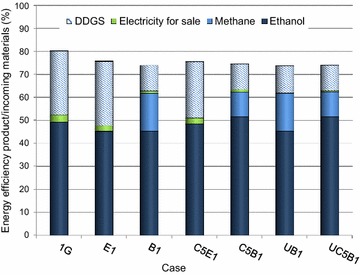
Energy efficiency for products/incoming energy

**Fig. 4:**
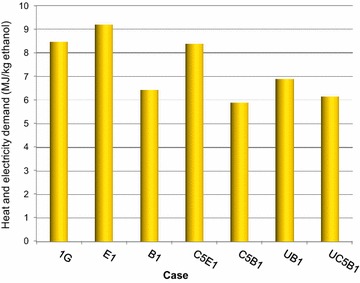
Heat and electricity required per kg ethanol produced

Only the excess electricity that can be sold was regarded as a product when calculating the energy efficiency. However, the total amount of electricity produced was considered in the economic assessment, since the income from green electricity certificates can be included in the profit. The energy efficiency and the net heat and electricity required to produce 1 kg of ethanol are presented in Figs. 3 and 4.

Figure 3 shows the energy efficiency for each product. The total energy efficiency for the products in the integrated cases was in the range of 74–76 %. The 1G case had a somewhat higher total energy efficiency of 80 %. The variation between the integrated cases is mainly due to the energy efficiency of the co-products. The higher energy efficiency for ethanol in the C5B1 case than in the C5E1 case, in spite of the fact that equal amounts of ethanol were produced, is due to the fact that natural gas had to be added in the latter case.

The total heat and electricity demand for the process per kg ethanol produced is shown in Fig. 4. The cases including evaporation (1G, E1, and C5E1) had a higher energy demand per kg ethanol produced than the cases without evaporation (B1, C5B1, UB1, and UC5B1). This was mainly due to the higher energy input in the evaporator trains and in the dryer. In all the cases including evaporation, extra energy in the form of natural gas had to be supplied to the process.

### Economics

**Fig. 5:**
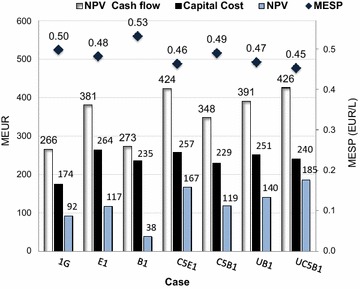
NPV for the capital cost, the cash flow and the overall revenue, and the MESP. The capital costs are presented as *black bars*, the NPV for the cash flow as *white bars*, overall revenue (total NPV) as *light blue bars* and the MESP as *dots*

**Fig. 6:**
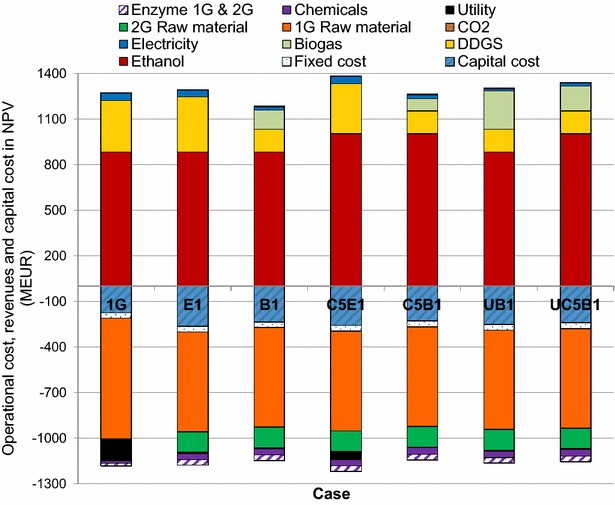
NPV for the capital costs, the fixed- and variable-operational costs and the revenues from the product

NPV was used to evaluate the profitability of the simulated cases, using a discount rate of 11 % and an investment lifetime of 20 years. The NPV, the minimum ethanol selling price (MESP), and the NPV, broken down into capital cost and operational cost and revenues for the cash flow, are shown for the seven cases in Figs. 5 and 6.

It can be seen from Fig. 5 that all the cases studied exhibited positive NPVs (light blue bars), and that the NPV increased considerably with C5 fermentation and biogas upgrading to vehicle fuel quality (C5E1, C5B1, UB1, and UC5B1) compared to the corresponding C6 fermenting case. However, the E1 case resulted in a much higher NPV than the B1 case, due to the larger amount of DDGS produced. The difference in NPV between cases C5E1 and C5B1 decreased, compared with cases E1 and B1, due to the fact that natural gas had to be utilized in the C5E1 case, as less residual sugars were available for anaerobic digestion. The 1G plant showed a lower NPV than the E1 plant. These results are in accordance with results presented by Dias et al. [4], showing that the internal rate of return (IRR) was higher with pentose fermentation than with biogas production from the pentoses in the integrated cases. In the present study, biogas upgrading to vehicle fuel quality was also investigated. It was shown that upgrading the biogas to vehicle fuel quality increased the NPV and IRR substantially for cases B1 and C5B1, due to that the price of biogas could be increased from 33 to 67 EUR/kWh (the revenue for the biogas increased by 50 % after upgrading to vehicle fuel quality Fig. 6). This made the UB1 and UC5B1 cases more profitable than the case including only C6 fermentation and evaporation (E1 and B1). However, the biogas yield is a very important factor in this case.

Figure 5 also shows the MESP for the different cases. The MESP was found to be between 0.46 and 0.53 EUR/L, and decreased with C5 fermentation and biogas upgrading to vehicle fuel quality, compared to the corresponding C6 cases. The NPV is slightly higher for the C5B1 case than the E1 case, while the MESP is lower for the E1 case than for the C5B1 case; this is because a larger amount of co-products is produced in the E1 case (Fig. 6). The NPV and MESP were higher for the 1G case than the E1 case because natural gas had to be added in the 1G case. Also, a somewhat higher amount of DDGS was produced in the E1 case because some of the C5 sugars will end up in the DDGS. The NPV and the MESP for the 1G case does not correspond exactly to a commercial 1G plant since e.g., a more advanced combined head and power (CHP) plant configuration was used in the model, for comparison with the integrated cases, than that required for a stand-alone 1G plant. A survey performed for dry-mill corn to ethanol plants in U.S (in consistent years 2002 dollar) showed in general lower values for the capital cost than for the 1G plant in the present study [27].

The capital cost in the 1G case was about 66–76 % of the capital cost in the integrated cases (Fig. 5), indicating that the investment risk would be lower in the 1G case than in the integrated cases. However, it is found that increasing the scope of the substrate and products increased the revenue in all but the B1 case, compared with the 1G case. Figure 5 can also be useful when discussing the possibility of expansion, for example, adding a 2G process line to an existing 1G plant. However, the cost of changing the process from 1G to 1G + 2G must be taken into consideration, and this may vary depending on the configuration of the 1G plant.

### Sensitivity analysis

Sensitivity analysis was carried out to assess the effect on the NPV of changes in the process conditions, the cost of raw materials, and the price of the products, as well as variations in the discount rate. The NPV for discount rates of 5, 11 % (base case), and 14 %, the MESP and the IRR are given for the seven cases and the six supplementing cases at a reduced biogas yield [60 % of the theoretical value of 0.25 kg methane/kg chemical oxygen demand (COD)] in Table 2. The biogas yield will be of great importance for the feasibility when investigating if biogas or DDGS should be produced from the C5 sugars.

**Table 2:** NPV, MESP, and IRR for all the cases

	NPV discount rate 11 % (MEUR)	NPV discount rate 5 % (MEUR)	NPV discount rate 14 % (MEUR)	MESP (EUR/L ethanol)	IRR (%)
1G	92	242	47	0.50	19
E1	117	332	53	0.48	17
E2	95	298	35	0.50	16
B1	38	192	−8	0.53	13
B2	−1	132	−41	0.56	11
C5E1	167	406	95	0.46	20
C5E2	154	387	85	0.47	20
C5B1	119	315	60	0.49	18
C5B2	90	271	36	0.51	17
UB1	140	360	74	0.47	19
UB2	71	250	17	0.51	15
UC5B1	185	426	114	0.45	22
UC5B2	134	344	72	0.48	19

NPV in the base case scenario and at higher and lower discount rates, together with the MESP and the IRR, for the seven cases studied, at 60 and 80 % of the theoretical biogas yield. Cases with 60 % biogas yield are indexed with “2” (E2, B2, C5E2, and C5B2)

The NPV decreased in cases E2 and B2 when the biogas yield was reduced to 60 % of the theoretical (0.25 kg methane/COD) (Table 2). In case B2, a negative NPV was found when the biogas yield was decreased to 60 %. The difference in NPV between cases E2 and B2 also increased when the biogas yield was decreased, as the profit from selling the biogas was lower in the B2 case, while it was unaffected in the E2 case (as no biogas was sold). The change in NPV due to lower substrate conversion in anaerobic digestion indicated that it is important to study the biogas process in the biorefinery more closely. In the cases including C5 fermentation (C5E2 and C5B2), the difference in NPV was smaller when a reduced biogas production was assumed, since less C5 sugars were available for anaerobic digestion. Nonetheless, the C5E1 case had a higher NPV than the C5B1 case. However, the NPV also shows that the amount of biogas produced is more important in the C6 fermentation cases than in the combined C5 and C6 sugars (C5&C6) fermentation cases. With regard to the IRR, it was found that cases C5E1, C5E2, UB1, UC5B1, and UC5B2 exhibited a higher IRR than the 1G case, implying that combined C5&C6 fermentation and biogas upgrading to vehicle fuel quality are important when considering an integrated plant. However, all cases except B2 exhibited an IRR above 11 %.

The results in Table 2 also show that at a discount rate of 5 %, the NPV increased by between 130 and 410 % compared with the corresponding base case scenarios, and decreased by between 40 and 120 % at a discount rate of 14 %. The trends for the NPV at the higher and lower discount rates mainly followed the trends in the NPV for the base case scenarios. However, a higher cash flow will have a greater impact on the financial results when the discount rate is altered, compared with the corresponding base case scenario. Therefore, the E1 case, for example, will exhibit a higher NPV than the C5B1 case at a 5 % discount rate, and the 1G case a higher NPV than the E2 case at a 14 % discount rate.

**Fig. 7:**
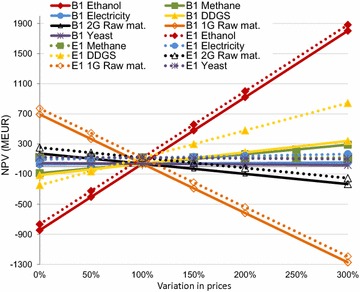
The effects of costs and prices variations on the NPV for cases B1 and E1

**Fig. 8:**
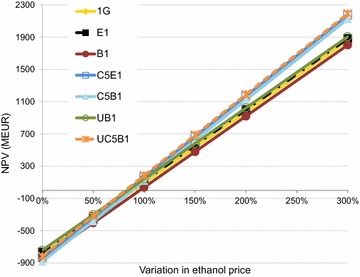
NPV for each case at different ethanol prices

**Fig. 9:**
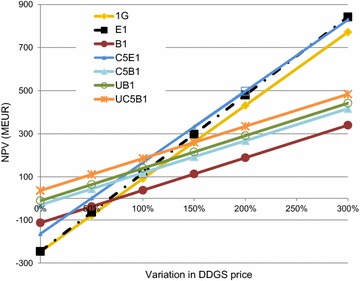
NPV for each case at different DDGS prices

**Fig. 10:**
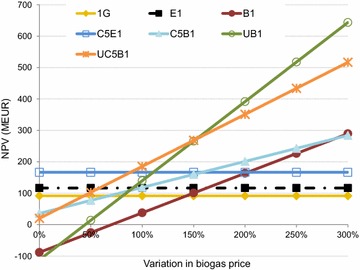
NPV for each case at different methane prices

To assess the impact of variations in the cost of raw materials and the price of products, a sensitivity analysis was conducted by varying the prices from 0 to 300 % of those used in the corresponding base case scenarios. The variations in the cost of raw materials and prices of products are compiled in Fig. 7 for cases B1 and E1, to visualize the magnitude of the different prices. The effects of varying the prices of ethanol, DDGS, and methane on the NPV for the various cases are presented in Figs. 8, 9, and 10.

It can be seen from Fig. 7 that the ethanol and 1G raw material prices have significant effects on the NPV, mainly because of the large quantities of raw material and ethanol involved. The effect on the NPV will also vary with the amount of co-products produced. It should also be pointed out that a price variation of +200 % for ethanol (1.1 EUR/L) is not as likely for example, as an increase of 200 % in the price of DDGS (0.55 EUR/kg), which has been found to fluctuate considerably.

The NPV decreased linearly with increasing cost of the raw material and yeast in all cases (data not shown). All the cases already showed a negative NPV at an increase in 1G raw material prices of 50 % (however, the effect of the possibility of a simultaneous increase in DDGS price was not accounted for). The amount of DDGS produced is also an important factor (see Figs. 6, 7), as the larger amount of DDGS produced in case E1 then in B1 gave a higher NPV at an increased DDGS price. The NPV decreased to zero in all cases when the price of 2G raw material was increased by 250 %. When the electricity price was varied, the largest effect on the NPV was seen in the scenarios where the most of the materials were incinerated, and therefore produced more electricity. The E1 case exhibited a lower NPV than the B1 case when the electricity price was decreased. However, all seven cases showed a positive NPV, even when the electricity price was set to zero.

The NPV increased in all cases with increasing ethanol price; the highest NPVs being seen for cases C5E1 and UC5B1 (Fig. 8). At an ethanol price of 110 % the NPV became higher for the C5B1 case than for the UB1 case, indicating that at a slightly higher ethanol price, C5&C6 fermentation will be more beneficial than upgrading the biogas to vehicle fuel quality in the B1 case. At an ethanol price of 80 % of the base case price all the cases showed a negative NPV.

It can be seen in Fig. 9 that NPV increased in all cases when the price of DDGS was increased, since DDGS was produced in all cases. The increase in NPV was steepest in the G1, E1, and C5E1 cases, as the amount of DDGS produced in these cases was largest. The E1 case exhibited a higher NPV than the C5E1 case at about 300 % higher DDGS price than in the base case scenario, since slightly more DDGS was produced in the E1 case. When the price of DDGS was increased by more than 50 %, the 1G case exhibited a higher NPV than all the cases that were designed to produce biogas from the thin stillage.

Cases B1 and C5B1 showed a higher NPV than cases E1 and C5E1 when the price of biogas was increased by 50–100 % of the base case price (Fig. 10). The cases including evaporation (E1, C5E1, and G1) were not affected by these price changes as the biogas produced in the plants was used internally, and no internal price was assigned to that biogas. In cases where natural gas must be purchased, the price of natural gas was assumed to be constant. However, the production of biogas is important as it reduces the amount of external fuel required. All cases except B1 and UB1 showed a positive NPV, even when no revenue was included for the biogas produced in the process.

## Conclusions

When designing an integrated 1G + 2G plant, one of two main configurations can be chosen: that producing biogas or that producing DDGS. The choice depends mainly on the market for DDGS and biogas at the location of the plant. Since the prices of both DDGS and biogas have been fluctuating a great deal in recent years, a detailed market analysis must be performed before making any decisions. However, in the current study, a large amount of DDGS and combined C5&C6 fermentation were found to be more promising than biogas production if the biogas was not upgraded to vehicle fuel quality. Furthermore, if legislation prevents the production of DDGS that includes genetically modified yeast, a separation step could be included in the 1G process before C5&C6 fermentation to separate the solids from the liquid fraction. The solids can then be utilized for DDGS production without being mixed with the yeast. A decrease in the biogas yield from 80 to 60 % also largely affects the NPV. Therefore, it is important to perform more detailed experiments on biogas production from 1G + 2G substrates.

## Methods

### Simulation tools and overall process modeling

The integrated plant was modeled assuming a 1G raw material loading of 360,000 tons dry grain per year and a 2G raw material loading of 180,000 tons dry wheat straw per year. These raw material loadings correspond to an estimated annual ethanol production of 200,000 m^3^, assuming C6 fermentation only. In some of the simulated cases, C5 fermentation was also considered, which increased the annual ethanol production to approximately 230,000 m^3^. It was assumed that the plant was in operation 8000 h per year, and could be managed by 28 people. One 1G case and six integrated 1G + 2G cases were modeled. In the integrated cases, ethanol, DDGS, and biogas production from the C5 sugars were investigated, as well as biogas upgrading to vehicle fuel quality. A sensitivity analysis was also performed for the six integrated cases to assess variations in the biogas yield which increased the investigated configurations to another six supplementary cases.

**Fig. 11:**
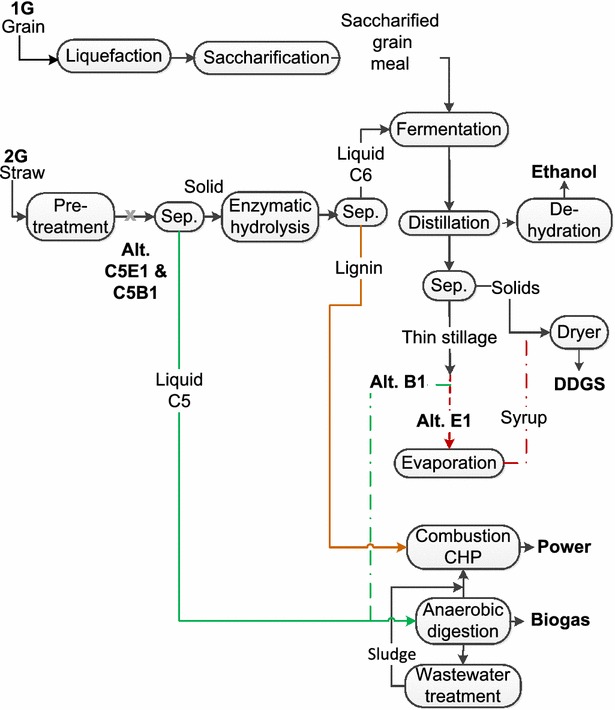
Schematic overview of the 1G + 2G process and alternative configurations

An overview of the process is shown in Fig. 11, and further details are provided in Section “Case description” below.

Simulations were performed with the flow sheeting program Aspen Plus (version 8.2 from Aspen Technology Inc., Massachusetts, USA). Data for biomass components such as cellulose and lignin were retrieved from the National Renewable Energy Laboratory (NREL) database developed for biofuel components [28]. The NRTL-HOC property method was used for all units except in the heat and power production steam cycle, where STEAMNBS was used. The simulation models were further developments of previous work by Wingren et al. [29, 30], Sassner and Zacchi [31] and Joelsson et al. [32]. Heat integration was implemented as described previously [32] using Aspen Energy Analyzer (version 8.2). The results from Aspen Plus were implemented in APEA, and were used together with vendors’ quotations to evaluate the capital and operational costs. Further details on the Aspen Plus modeling can be found in a previous publication [33].

## Experimental setup and model assumptions

### Raw materials

Dry-milled WBG (1G material) with a wheat to barley ratio of 80:20 was kindly provided by Lantmännen Agroetanol. The WBG had a dry matter (DM) content of 89 %, which contained 76 % starch. The 2G material (wheat straw) used in the experiments was obtained from Johan Håkansson Lantbruksprodukter (Lunnarp, southern Sweden). The straw was first cut with a knife mill and then sieved to obtain particles of 2–10 mm. The straw consisted of approximately 91 % DM. The NREL method for determining structural carbohydrates and lignin content in biomass [34] was used to determine the composition of the straw.

The components of the dry raw materials are given in Table 3. Since all the components were not determined in the analysis, an average raw material composition was enlisted for both the 1G and the 2G materials, as this was required to close the mass balances for the simulations in Aspen Plus. The composition of the WBG was complemented with data estimated from the literature [35, 36] and previously performed studies by Erdei et al. [23, 25]. The average wheat straw composition was supplemented with results from studies performed by Erdei et al. [23] and Linde et al. [37].

**Table 3:** Raw material composition obtained from experiments and the compositions used in Aspen Plus

	Straw (% of DM)	Straw comp. used in Aspen Plus (% of DM)	WBG comp. used in Aspen Plus (% of DM)
Glucan^a^	42.3	42.2	6.8
Starch	0.0	0.0	76.0
Xylan	27.4	26.7	4.9
Arabinan	3.6	3.1	0.0
Lignin	19.3^b^	15.5	0.5
Ash	1.4	0.16	1.6
Acetate	n.a.^c^	1.5	0.0
Extractives	n.a.	10.2^d^	0.0
Protein	n.a.	0.7	7.9
Fat	n.a.	0.0	2.3

^a^Cellulose as glucan. ^b^ of which acid-soluble lignin was 1.1 wt%, ^c^ not analyzed, ^d^ of which 5 wt% was non-volatile and 5.2 wt% semi-volatile

### General experimental procedure

The experimental procedure consisted of steam pretreatment of dilute-H_2_SO_4_-impregnated wheat straw, followed by liquid–solid separation of the material. The liquid part, containing most of the C5 sugars from the hemicellulose, was not processed further in the experimental study; however, in the simulations it was used in anaerobic digestion when C5 fermentation was not considered. The solid material was subsequently treated by EH. After EH, filtration was performed to separate the liquid fraction from the solid fraction, containing mainly lignin. The liquid fraction was then fermented with pre-liquefied and saccharified WBG. The proportion of straw to grain was 1:2.

### Steam pretreatment

An aqueous solution of 0.2 wt% H_2_SO_4_ was used to impregnate the wheat straw for 1 h, using a liquid to dry straw weight ratio of 20:1. The impregnated straw was pressed to an average DM content of 53 % before being stored overnight in a sealed plastic bucket until pretreatment. Pretreatment was performed in a 10-L steam pretreatment unit described elsewhere [38], for 10 min at 190 °C, according to the findings of Linde et al. [37]. The SPWS slurry was thoroughly mixed before being stored at 4 °C until it was pressed to a water-insoluble solid (WIS) content of 37 %.

Triplicate samples were used to determine the DM and WIS contents of the SPWS. Standardized analytical NREL procedures [39] and [34] were used to determine the total soluble sugars and degradation products in the liquid fraction of the SPWS and the carbohydrates and lignin in the solids.

The pretreatment step was modeled with an RStoic reactor in Aspen Plus. The reactor was assumed to be operated as a continuous reactor into which 20-bar steam was injected at 190 °C. To account for heat losses and the void of the reactor, the steam consumption was increased by 10 % compared to an adiabatic unit. The outgoing material was assumed to be cooled by two-step flashing, at 4 and 1 bar. The main part of the flashed steam, which contains volatile compounds formed during pretreatment, was condensed and cooled before being fed to an anaerobic digestion unit and then a waste-water treatment unit. Part of the steam was recirculated and used to preheat the incoming wheat straw.

The composition of the SPWS and the recovery factors for carbohydrate and lignin used in Aspen Plus are given in Table 4. The recovery factors used in Aspen Plus are given for the liquid and WIS fractions. The residual carbohydrates not given were modeled as degradation products assumed to be present in the liquid fraction.

**Table 4:** Raw material composition of SPWS and recovery factors used in Aspen Plus

Raw material composition SPWS		Recovery factors used in Aspen Plus		
			Solid fraction (WIS) (%)	Liquid fraction (%)
Total solids (%)	17.0			
WIS content (%)	12.0			
Solid fraction (% of WIS)				
Glucan	59.5	Glucan	85.3	6.6
Xylan	4.4	Xylan	1.0	59.1
Arabinan	0.6	Arabinan	10.3	84.6
Lignin	24.9	Acetate	10.0	90.0
Ash	5.8	Lignin	97.5	2.5
Liquid fraction (g/L)				
Sugars^a^				
Glucose	7.0	Ash	80.0	20.0
Xylose	40.4	Extractives	0.0	100.0
Arabinose	4.7			
By-products				
Furfural	3.3			
HMF	0.3			
Acetic acid	3.9			

^a^Total sugars containing both monomer and oligomer sugars

### Enzymatic hydrolysis

#### WBG hydrolysate

Two-step enzymatic hydrolysis (liquefaction and saccharification) was used to produce the WBG starch hydrolysate used in fermentation. Hydrolysis was performed in a 10-L evaporator (Büchi Labortechnik AG, Flawil, Switzerland) with a working weight of 7 kg in each batch. The WBG was mixed with water to achieve a DM of 35 % and then liquefied by thermostable α-amylases (Termamyl SC; Novozymes A/S, Bagsværd, Denmark) at 85 °C, pH 5.5 for 3 h, using an enzyme dosage of 0.5 g enzyme/kg DM WBG. After 3 h of liquefaction, the temperature was reduced to 60 °C, the pH was adjusted to 4.2, and amyloglucosidase (Spirizyme Fuel; Novozymes A/S) was added at a dosage of 1 mL/kg DM in order to saccharify the liquefied WBG for 24 h. To obtain a homogeneous material, all the batches were combined into one large batch. The WIS content and the carbohydrate concentrations in the hydrolyzed WBG were determined before fermentation.

#### SPWS

EH was performed on duplicate samples of the solid fraction of the pressed SPWS in a 30-L fermentor vessel (Bioengineering AG, Wald, Switzerland), using a total working weight of 15 kg. The pressed SPWS was diluted to a WIS concentration of 10 % before hydrolysis was performed with a Cellic CTec3 cellulase enzyme preparation (Novozymes A/S Bagsværd, Denmark) at an enzyme loading of 10 filter paper units (FPU)/g WIS. Hydrolysis was carried out for 96 h, at 45 °C, pH 5.0, and at a stirrer speed of 500 rpm. The pH was adjusted to 2 after EH to precipitate colloidal low molecular weight lignin and improve the separation of the solid and liquid fractions. The hydrolysate was then centrifuged at 4000 rpm for 10 min in several batches, which were mixed into one homogeneous batch. The pH of the hydrolysate was adjusted to five by the addition of NaOH, before fermentation. The washed solid fraction was analyzed to determine the carbohydrate, lignin, and ash contents.

### Fermentation and yeast cultivation

#### Experimental procedure

Fermentation was carried out in duplicate batches using the 30-L fermentor vessels described above with a total working weight of 21.5 kg. The hydrolysate from the EH of the SPWS was autoclaved in 5-L bottles at 120 °C for 20 min before being added to the fermenters. A nutrient solution, consisting of a sterilized solution of 10.0 g (NH_4_)_2_HPO_4_ in water, was added to the vessel to give a final concentration of 0.5 g/L. The pH was maintained at 5 during fermentation with a 10 % NaOH solution, and fermentation was carried out for 120 h at 30 °C with stirring at 500 rpm. A cell suspension of conditioned Ethanol Red yeast (Fermentis, Marcq en Baroeul, France) was added to the fermentation vessel at a concentration of 3 g/L in the final liquid fraction to initiate fermentation of the EH supernatant from the SPWS. The glucose from the EH supernatant of the SPWS was depleted after 8 h, at which time feeding of the saccharified WBG solution was started and continued until 72 h. The feeding rate was 200 mL WBG/h, corresponding to the addition of approximately 50 g glucose to the fermentation vessel per hour. A commercial C6-fermenting yeast was used in the experiment since the main focus of the study was the hydrolysis and integrated fermentation of starch and cellulose material in a process development unit. However, lab-scale experiments on integrated grain and wheat straw fermentation using a co-fermenting strain have been performed, showing good conversion factors for both xylose and glucose to ethanol [24].

#### Simulations

As earlier lab-scale experiments had shown good conversion of both C5 and C6 to ethanol, C6 fermentation as well as co-fermentation of C5&C6 was modeled. The solid–liquid separation step that was carried out in the experiments after pretreatment was omitted in the simulated C5&C6 co-fermentation cases. In the Aspen Plus model, it was assumed that both C6- and combined C5&C6-fermenting yeast were cultivated on glucose from starch in a separate tank. The yeast production yield was assumed to be 0.5 g dry biomass/g glucose, and the pitch rate 3 g/L. It was assumed that commercially available enzymes were purchased for the hydrolysis of WBG and SPWS. EH and fermentation were modeled with RStoic reactors in Aspen Plus. The residence times were set to 24 h for the EH of SPWS, 28 h for the liquefaction and saccharification of WBG and 96 h for fermentation in the integrated cases. The cleaning and refilling times were set to 8 h, in the liquefaction and saccharification stage of WBG and to 12 h in the hydrolysis of the SPWS and the fermentation stages.

The overall process conversion factor of glucose to ethanol from the raw materials was set to 0.80 based on the experimental results after withdrawing the sugar needed for yeast cultivation, since yeast cultivation was not included in the experiments. In the combined C5&C6 fermentation, the xylose to ethanol conversion factor was set to 0.9 in the fermentation step, and a xylan to xylose conversion factor of 0.95 was used in EH, which was higher than that achieved in the experiment, but should be the aim in a future industrial plant. It was assumed in the simulations that 50 wt% of the xylan in the WBG was converted to xylose, due to hemicellulolytic activity in the amyloglucosidase preparation [40]. The final ethanol concentrations in the broths obtained in the simulations of the fermentation of C6 and combined C5&C6 sugars in the integrated plants were 9.6 and 10.8 wt%, respectively. The final ethanol concentration in the simulation of the 1G stand-alone plant before the distillation step was 11 wt%.

#### Yield calculation

The overall ethanol yield (*Y*_*p*_) from the glucose in the raw material was calculated from Eq. (1),

1 $$Y_{p} = \frac{{E_{e} }}{{E_{t} }},$$

where *E*_*t*_ is the total theoretical amount of ethanol that could be produced from the raw materials used in the experiments, based on their composition, and *E*_*e*_ is the amount of ethanol produced in the experiments. The theoretical amount of ethanol that could be produced from xylose in the raw materials, was calculated in the same way as for glucose.

### Distillation

It was assumed that a 25-stage, low-pressure stripper column followed by a 35-stage, high-pressure rectifying column, were used in the distillation process to concentrate the fermentation broth to 92.5 wt%. The ethanol stream was then dehydrated with molecular sieves to 99.5 wt%. A top-stage pressure of 0.3 bar and a Murphree efficiency of 50 % were assumed in the stripper column, and 1.3 bar and a 75 % Murphree efficiency in the rectifying column. The pressure was kept low in the stripper column to avoid gluten fouling, which can occur at higher temperatures. The rectifier was equipped with a partial-vapor condenser, where the heat from condensation was used to heat the stripper column. The overhead vapor from the rectifier was superheated and then concentrated to about 99.5 % in molecular sieve dehydration columns before cooling and storage. The reject stream from the molecular sieves was mixed with the condensed overhead vapor from the stripper column before being fed to the rectifier. The ethanol concentration in the rectifier feed was approximately 55 wt%, and the mass reflux ratio about 2.1.

### Solids separation

Thick stillage from the stripper columns, with a DM content of between 10 and 15 wt% (depending on the case), was filtered to attain a wet cake, consisting of solid particles with 45 wt% DM, and a thin stillage. The thin stillage contained between 6 and 11 wt% soluble DM. The filter unit was assumed to have a retention of solid particles of 95 % [41]. The thin stillage was then processed in two alternative ways. It was either sent to an evaporator, where it was concentrated to syrup before being mixed with the wet cake, and the overhead vapor was condensed and partly recirculated in the process. In the other alternative, the thin stillage was partly recirculated and the rest was subjected to anaerobic digestion, and only the wet cake was dried.

### Evaporation

Evaporation was modeled as a five-effect, forward-feed system. The boiling point elevation was accounted for using the expression derived by Larsson et al. [42]. Steam at 4 bar was used as heating medium and applied to the first evaporator operating at a pressure of 3 bar. The liquid fraction from the last evaporator unit was assumed to have a DM content of 60 wt%, and to leave the system at 0.2 bar. The condensed overhead vapor that was not recirculated was anaerobically digested.

### Drying

A steam dryer was used to dry the incoming wet cake or the mixture of the wet cake and syrup to produce DDGS. The incoming material was dried with 4 bar superheated steam at 200 °C. Most of the outgoing steam from the dryer (90 %) was superheated and recirculated back to the dryer, and the remaining 10 % was condensed and fed to the anaerobic digestion stage. The outgoing solids from the dryer were assumed to have a DM content of 88 wt%.

### Anaerobic digestion

The liquid fraction after pretreatment, the rectifier and the thin stillage, together with the condensed steam from the dryer, pretreatment and evaporation stages, were anaerobically digested for the production of biogas. Because experimental data regarding the exact substrate mixture are limited, some assumptions had to be made for the anaerobic digestion step, which could lead to some uncertainty in the model. However, Nkemka and Murto [43] showed that 0.19 kg methane could be produced per kg COD from steam-pretreated, enzymatically hydrolyzed wheat straw in trial batch experiments. According to a review by Wilkie et al. [44] on ethanol stillage from conventional and cellulosic feedstock, stillage from cellulosic feedstock could result in a methane yield of 0.21 kg methane/kg COD, resulting in the removal of about 84 % of the COD. Anaerobic digestion was modeled as follows, assuming a theoretical methane production of 0.25 kg methane/kg COD. An RStoic reactor was set up in Aspen Plus in which total degradation of all the components in the incoming feed to the reactor was assumed, and the amount of oxygen required was calculated. A base case scenario was then set up assuming a COD removal of 80 %, which was multiplied by the theoretical methane production, resulting in a methane yield of 0.20 kg methane/kg COD (In addition a COD removal of 60 % was also used, to simulate a lower biogas production). The outgoing biogas stream consisted of 50 % methane, 46 % carbon dioxide, and 4 % water. The sludge generated in the anaerobic digestion step was combusted. Pressure swing absorption was used to upgrade the biogas to vehicle fuel quality [45].

### The stand-alone 1G plant

The residence time in the fermentation step was set to 55 h, and the ethanol concentration in the broth after fermentation was set to 11 wt%. The cleaning cycle time in the fermentation step was the same as in the integrated cases. The raw material loading was increased to 55 ton DM/h to achieve the same ethanol production as in the integrated C6-fermenting cases. The CHP plant (described in Section “Energy supply”*)* was used in the 1G plant. (The CHP unit included a high-pressure boiler, which means that the capacity of the steam supply system was higher than in a normal stand-alone plant utilizing natural gas, which is common for corn-based ethanol plants operating in the USA today.)

## Energy supply

The heat and electricity required in the process were supplied by a co-located CHP. Superheated steam at 90 bar and 470 °C was produced by a boiler fueled by the solid residues, mainly lignin, generated in the solid separation step after the EH of SPWS, together with biogas and sludge produced in the anaerobic digestion step. If the process did not generate sufficient material for combustion, surplus energy in the form of natural gas (methane) was included in the simulations. The 1G case was run entirely on natural gas. Surplus energy could also be obtained by burning straw or other residual material instead of natural gas, since both systems exist. A steam turbine system was connected to the boiler to generate electricity. High-temperature steam required in the pretreatment and the drying steps was withdrawn at 20 bar, and medium-temperature steam was withdrawn at 4 bar to supply the rest of the process with heat. The isentropic and the mechanical efficiencies for the turbines were set to 90 and 97 %, respectively. The electricity demand of the plants was calculated from evaluations made using APEA and estimates specified in the manufacturers’ quotations. The electricity requirement of the process was then subtracted from the total amount produced in the plant, and any surplus was sold to the grid.

The energy content was calculated using the LHV of the materials, obtained from the values of heat of combustion calculated in Aspen Plus. The LHV for the incoming materials WBG and straw were 15 and 19 MJ/kg DM, 11 MJ/kg DM for the enzymes, and 27, 50, and 16 MJ/kg DM for the outgoing products, ethanol, methane, and DDGS, respectively. The energy required for harvesting and transporting the raw materials an average distance of 50 km to the plant was set to 0.03 MJ/MJ dry biomass [46]. The DM contents of grain, straw, and DDGS were set to 89, 91, and 88 %, respectively.

The energy efficiency of the outgoing products was calculated as the energy flow (in MW) in the products divided by the energy flow into the process [shown in Eq. (2)]. The incoming energy included the energy in the raw materials, enzymes, surplus natural gas, and the energy required for harvesting and transportation.

2 $${\text{Energy efficiency}} = \frac{\text{Energy in product}}{{{\text{Energy in rawmaterial}} + {\text{Enzyme}} + {\text{Natural gas}} + {\text{Harvests }}\& {\text{transportation}}}}$$

## Case description

Seven cases were simulated to obtain the NPV and energy demand of different process alternatives. The first case was a stand-alone 1G plant, and the six following cases were modeled as integrated 1G + 2G plants. The cases are listed in Table 5 and illustrated in Fig. 11.

**Table 5:** Descriptions of the cases simulated

Case	1G^a^	E1^a^	B1^a^	C5E1^a^	C5B1^a^	UB1^a^	UC5B1^a^
C6 fermentation only	x	x	x			x	
C5&C6 fermentation				x	x		x
Evaporation thin stillage	x	x		x			
Biogas from thin stillage			x		x	x	x
Upgrading of biogas to vehicle fuel quality						x	x

^a^The cases are denoted with “1” to indicate that the biogas yield was 80 % of the theoretical value (cases with a biogas yield of 60 % will be denoted with the number 2)

The first case (1G), a stand-alone 1G plant, was modeled to produce the same amount of ethanol as the integrated C6-fermenting cases. Cases E1 and B1 were modeled using the experimental results obtained with a C6-fermenting yeast. In E1, the thin stillage generated after the separation step of the stillage from the distillation was concentrated by evaporation. The syrup generated was subsequently added to the solid fraction and dried to produce DDGS. In B1, the thin stillage was sent directly to the anaerobic digestion step to produce biogas, and only the solid fraction from the stillage was used for DDGS production. In cases C5E1 and C5B1, co-fermentation of C5&C6 sugars was assumed, and the cases were modeled as for E1 and B1, respectively, from the separation step after distillation and downstream. The biogas yield was 80 % of the theoretical (0.25 methane/COD) in all the seven cases. In the C5E1 and C5B1, it was assumed that the DDGS could be sold as animal feed. However, this will depend on the yeast used and/or future regulations regarding genetically modified substances in animal feed. In the UB1 and UC5B1 cases, the surplus biogas was upgraded to vehicle fuel quality and thereby also the price of the biogas was increased. An overview of the different process alternatives can be seen in Fig. 11.

## Cost calculations

Vendors’ quotations and APEA were used to calculate the capital cost of a plant located in Sweden. The energy and mass balances obtained from Aspen Plus were used to size the equipment. The costs for the following equipments were estimated based on vendors’ quotations; boiler, dryer and pelletizing equipment, molecular sieves, filter presses, anaerobic digestion, and pretreatment units. The chemical engineering plant cost index was used to update the prices to 2012 values. The price index for the calculations was based on 2012, and the construction time of the plant was 1 year. An exchange rate of 1 EUR = 9 SEK was used. The capital cost item included the cost of equipment and installation, civil construction, electricity installation, instrumentation, land, engineering, and fees associated with the investment cost. The operational cost item was based on Swedish conditions and separated into variable and fixed costs. Fixed costs included maintenance, insurance, working capital, and labor. The cost of working capital was determined according to recommendations in the literature [47], and was assumed to be equivalent to an interest rate of 11 %. The costs of chemicals and utilities are given in Table 6. The costs are average values estimated after personal communication with companies and open source references. The cost of the enzymes in the 2G process was set to 3.0 EUR per million FPU, while for the 1G process it was 3.33 EUR/kg enzyme solution. The cost of enzymes for the 2G process was estimated based on figures from Novozymes assuming a cost of enzyme to be about 0.5 $/gallon ethanol produced from cellulosic material [48]. The cost of the yeast license was set to 0.01 EUR/L ethanol produced. The selling price of DDGS was based on the cost of rape seed meal.

**Table 6:** Cost of raw materials, chemicals and utilities, and prices of products

Input	Cost/unit	Units
Wheat grain^a^	0.23	EUR/kg DM
Wheat straw^b^	0.09	EUR/kg DM
Yeast license	0.01	EUR/L ethanol produced
Enzyme 2G	3.00	EUR/10^6^ FPU
Enzyme 1G	3.33	EUR/kg enzyme solution
Sulfur dioxide^c^	0.17	EUR/kg
Phosphoric acid (50 %)^c^	0.56	EUR/kg
(NH_4_)_2_HPO_4_^c^	0.67	EUR/kg
MgCl_2_^c^	0.17	EUR/kg
Ammonia (25 %)^d^	0.22	EUR/kg
Antifoam^e^	2.22	EUR/kg
Utilities		
Process water^f^	0.16	EUR/kg
Cooling water^f^	0.02	EUR/kg
Natural gas^g^	33.00	EUR/MWh
Products		
Ethanol	0.56	EUR/L
Biogas, raw^g^	33.00	EUR/MWh
Biogas, upgraded to vehicle fuel quality^g^	67.00	EUR/MWh
DDGS^h^	0.27	EUR/kg dry
Electricity (spot and certificate price)^i^	0.67	EUR/MWh
Carbon dioxide^j^	0.003	EUR/kg

^a^[49], ^b^ [50]. ^c^ [51], ^d^ [52], ^e^ [53], ^f^ [54], ^g^ [45], ^h^ [55]^,^^i^ [56], ^j^ [57]

Equation (3) was used to calculate the NPV of the capital cost (*I*_0_) and the operational costs, called the cash flow (CF), for an investment with a lifetime (*n*) of 20 years. A discount rate (*r*_*d*_) of 11 % was set in the base case scenarios. A straight line depreciation schedule with a period of 10 years was chosen for the investment in fixed assets.

3 $${\text{NPV}} = I_{0} + \mathop \sum \limits_{n = 1}^{n} \frac{{{\text{CF}}_{n} }}{{(1 + r_{d} )^{n} }}$$

A MESP was calculated for the different cases by setting the NPV to zero. The MESP and the NPV were then used to evaluate the cases. The results of the calculations should primarily be used to compare the different cases with each other, and should not be taken as absolute values. A more detailed investigation is required for financial decisions.

## Sensitivity analysis

Sensitivity analyses were performed for variations in the cost of raw materials, the prices of products, the biogas yield, and the discount rate, in order to investigate their effect on the NPV. The biogas yield was decreased by changing the COD removal from 80 % of the theoretical value (0.25 kg methane/kg COD at 100 %), to 60 % (0.15 kg methane/kg COD). The cases with 60 % biogas yield were indexed with “2”. The cost of raw materials and the selling prices of products were varied from 0 to 300 % for each case, compared with the costs and prices used in the base case scenario. In addition, the effects of varying the cost of yeast per L ethanol produced were investigated. The costs and prices were varied separately, so synergy effects were not considered. The base case scenario prices are presented in Table 6. The discount rate (11 % in the base case scenario) was varied from 5 % (risk-free asset) to 14 % (higher expected return).

## Abbreviations

1G: First-generation
2G: Second-generation
1G + 2G: 1G and 2G
DDGS: Distillers dried grain with solubles
C5: Xylose
C6: Glucose
C5&C6: C5 and C6 sugars
MESP: Minimum ethanol selling price
NPV: Net present value
LHV: Lower heating value
IRR: Internal rate of return
DM: Dry matter
COD: Chemical oxygen demand
CHP: Combined heat and power
APEA: Aspen Process Economic Analyzer
FPU: Filter paper units
WBG: Wheat and barley grain meal
SPWS: Steam-pretreated wheat straw
WIS: Water-insoluble solids
NREL: The National Renewable Energy Laboratory
EH: Enzymatic hydrolysis
